# Supplementary material for: PARP1 orchestrates variant histone exchange in signal-mediated transcriptional activation
Source: EMBO Rep. 2013 Oct 22;14(12):1084–91. doi: 10.1038/embor.2013.164 (PMC3981078; doi:10.1038/embor.2013.164)
Supplement: Supplementary Information [file embor2013164s1.pdf]

Supplementary Information: O'Donnell et al.

Supplementary Materials and methods

Cell culture. HeLa cells were maintained in 10% DMEM containing 10% FCS. MCF10A cells were maintained in DMEM/F12 containing 5% horse serum, 20 ng/ml EGF, 10 μg/ml insulin, 100 ng/ml cholera toxin and 0.5 μg hydrocortisone. Mouse Embryonic Fibroblasts (MEFs) *Parp1-/-* and *Parp1+/+*, kindly gifted by Valerie Schreiber, were maintained in 10% DMEM containing 10% FCS. Polyclonal *Parp1*-/-[pIRESpuro3-PARP1(WT)] and *Parp1*-/-[pIRESpuro3-PARP1(E988K)] MEFs were created by transfecting *Parp1-/-* MEFs with pIRESpuro3-PARP1(WT) and pIRESpuro3-PARP1(E988K) (Patel et al., 2012) and selection for 14 days in media containing puromycin (5 μg/ml).

ChIP and re-ChIP assays. HeLa cells were treated with 1% formaldehyde for 10 minutes at room temperature before quenching with 0.125 M Glycine for 5 minutes. Cells were harvested in ice-cold PBS with complete protease inhibitors (Roche) and washed sequentially with Buffer I (10 mM HEPES pH 6.5, 0.5 mM EGTA, 10 mM EDTA, 0.25% Triton X-100) and Buffer II (10 mM HEPES pH 6.5, 0.5 mM EGTA, 1 mM EDTA, 200 mM NaCl) then resuspended in SDS lysis buffer (50 mM Tris pH 8.1, 10 mM EDTA, 1% SDS). Lysates were sonicated using a Bioruptor Sonicator to yield 200-600 bp DNA fragments. One quarter of a 10 cm dish was used per ChIP (1/40 dish used for core histone IP), diluted 1/10 in IP Dilution buffer (0.01% SDS, 1.1% TRITON-X-100, 1.2 mM EDTA, 16.7 mM TrisHCl pH 8.1, 167 mM NaCl) and incubated overnight at 4°C with either 1 μg anti-PARP1 (Santa Cruz #sc-25780), anti-poly(ADP-ribose)

(Enzo #SA-216; Santa Cruz, sc-56198), anti-NFI (Santa Cruz #sc-5567), anti-Histone H2A (Millipore #07-146; Abcam #18255), anti-Histone H3 (Millipore #04-928), anti-Histone H2A.Z (Abcam #ab4174), anti-POLR2A (Santa Cruz #sc-899), anti-p400 (Bethyl lab s#A300-541A; Santa Cruz #sc-66497), anti-INO80 (Abcam #118787), anti-acetylated histone H3 (acetyl Lys9) (Abcam #ab4441 or 1μg non-specific IgG (Millipore #12-370). Immunocomplexes were precipitated by incubation for 60 minutes with protein G-conjugated magnetic beads (Invitrogen) that had been pre-blocked by incubation with 2 μg salmon sperm DNA. Immunoprecipitates were washed sequentially with TSEI (20 mM, Tris pH 8.1, 2 mM EDTA, 150 mM NaCl, 1% Triton, 0.1% SDS,), TSEII (20 mM Tris pH 8.1, 2 mM EDTA, 500 mM NaCl, 1% Triton, 0.1% SDS), Buffer III (10 mM Tris pH 8.1, 0.25 M LiCl, 1 mM EDTA, 1% NP40, 1% DOC) and 1xTE before eluting in 10% Chelex (BioRad). Cross-links were reversed by heating to 99°C for 10 minutes, then treating with proteinase K for 3 hours at 65°C. Quantitative PCR was performed in at least duplicate, from at least two independent experiments, using Quantitect SYBR green PCR reagent (Qiagen) and a Rotorgene 3000 machine (Corbett Research) and the PCR primer pairs; human *FOS* "-130" (ADS1686/ADS1687), *FOS* "-3000" (ADS2810/ADS2811), *FOS* "+160" (ADS2151/ ADS2152) and *FOS* "+2000" (ADS2125/ADS2126) or mouse *Fos* promoter (ADS2770/ ADS2771). Results were analysed with Rotorgene 6.0 software (Corbett Research) relative to input using the standard curve method. Specific endogenous ChIP enrichments were all at least 3-fold over normal IgG ChIP, and at least 3-fold more than ChIP of an intronic region of the *SRF* gene "+5134" (ADS1684/ ADS1685). For re-ChIP, ~10^7 cells were used per IP with either anti-PARP1 or anti-NFI antibodies, and continued as above but adding protease

inhibitor cocktail (Roche) to all washes and keeping samples on ice at all times. After the 1xTE wash, immunoprecipitated complexes were twice eluted gently in 15 μl 10 mM DTT for 30 minutes at 37°C. Combined eluates were added to 1 ml 2nd IP buffer (20 mM Tris pH8.1, 2 mM EDTA, 0.1% SDS 1% Triton, 150 mM NaCl) and incubated with the 2nd antibody overnight. Samples washed and analysed as above.

Mono-nucleosome ChIP. HeLa cells (half 10 cm dish per IP) were treated with 1% formaldehyde for 10 minutes at room temperature before quenching with 0.125 M glycine for 5 minutes. Cells were washed 3 times in PBS before harvesting in ice-cold PBS. Cells were incubated in swelling buffer (25 mM HEPES pH 7.9, 1.5 mM $MgCl_2$, 10 mM KCl, 0.1% NP40, 0.5 mM PMSF) for 10 minutes and Dounce homogenised with 20 strokes. The cell pellet was resuspended in 10 volumes sucrose buffer A (0.32 mM Sucrose, 15 mM HEPES pH 7.9, 60 mM KCl, 2 mM EDTA, 0.5 mM EGTA, 0.5% BSA, 0.5 mM Spermidine, 0.15 mM Spermine, 0.5 mM DTT) and Dounce homogenised 20 strokes. The nuclear suspension was layered over an equal volume of Sucrose Buffer B (0.32 mM Sucrose, 15 mM Hepes pH 7.9, 60 mM KCl, 2 mM EDTA, 0.5 mM EGTA, 0.5 mM Spermidine, 0.15 mM spermine, 0.5 mM DTT) and pelleted. After washing with buffer NUC (0.34 mM sucrose, 15 mM HEPES pH 7.5, 60 mM KCl, 0.5 mM spermidine, 0.15 mM spermine, 0.15 mM beta-mercaptoethanol), nuclei were pelleted and resuspended in fresh Buffer NUC. $CaCl_2$ was added to 3 mM and 200 U micrococcal nuclease (MNase). After incubation at 37°C for 30 minutes, the digest was stopped by adding an equal volume of sonication buffer (90 mM HEPES pH 7.9, 220 mM NaCl, 10 mM EDTA, 2% Triton, 0.2% Na-Deoxycholate, 0.2% SDS, 0.5mM PMSF). Nuclei were

lysed by sonication and insoluble material discarded. Chromatin immunoprecipitation continued as for regular ChIP as above. Chromatin was digested to mono-nucleosome level as evidenced by agarose gel and ethidium bromide visualisation. Also, real-time quantitative PCR failed to amplify DNA surrounding the nucleosome.

Chromatin Accessibility by Real-Time PCR (CHART-PCR). HeLa cells (60 mm dish) were harvested in ice cold PBS. Cell pellets were lysed on ice for 5 minutes in 500μl CHART Lysis Buffer (10 mM Tris pH 7.4, 10 mM NaCl, 3 mM $MgCl_2$, 0.5% NP40, 0.15 mM Spermine, 0.5 mM Spermidine). Nuclei were isolated by centrifugation, and then washed in 150 μl Buffer A (100 mM NaCl, 50 mM Tris pH8.0, 3 mM $MgCl_2$, 0.15 mM Spermine, 0.5 mM Spermidine). After pelleting, nuclei were re-suspended in 100 μl buffer A supplemented with 1 mM $CaCl_2$ and 12 μl aliquots made to tubes containing various dilutions of DNaseI ranging from 0 to 2 U. DNA was digested at 37°C for 5 minutes and reaction was stopped by adding 5 mM EDTA, 10 μg proteinase K and 1% SDS and incubating at 55°C for 2 hours. DNA was cleaned using QiaQuick PCR cleanup columns (Qiagen) and real-time PCR used to measure the yield of product, which is inversely proportional to the accessibility of DNaseI to the region. Digest conditions were optimised to digest only the active regions of DNA and not a negative control region of the *HBB* gene using the following primers: (ADS2831/ADS2832). Results are presented as % DNase accessibility, which was calculated as the % change in PCR product levels, after digestion, relative to an undigested chromatin sample.

Quantitative RT-PCR. Total RNA was harvested using a RNeasy kit (Qiagen). 40 ng RNA was used in a one-step RT-PCR reaction using Quantitect SYBR green reagent (Qiagen) using the following primer pairs: human *FOS* (ADS1690/ADS1691) or mouse *Fos* (ADS2760/ADS2761); *H2A.Z* (ADS2233/ADS2234); *p400* (ADS2123/ADS2124), *INO80* (ADS2758/ADS2759), *PARP1* (ADS2262/ADS2263), *PARP2* (ADS 2756/ADS2757), or *GAPDH* (ADS2184/ADS2185) primers.

Western Blot Analysis. Western blotting was carried out using primary antibodies; anti-PARP1 (Santa Cruz #sc-25780), anti-PARP2 (Proteintech #55149-1-AP), anti-poly (ADP-ribose) (Enzo #SA-216), anti-FOS (Santa Cruz #sc-7202), anti-ERK2 (Santa Cruz #sc-154), anti-phosphoERK (Cell Signaling #9106), anti-ELK1 (Epitomics #1277-1), anti-phosphoELK1 (Cell Signaling #9181), anti-Histone H2A.Z (Abcam #ab4174), anti-p400 (Bethyl labs#A300-541A), anti-INO80 (Abcam #118787), anti-NFI (Santa Cruz #sc-5567), and anti-p300 (Santa Cruz #sc-584). Data were visualised using infra-red dye conjugated secondary antibodies detected by a LiCor Odyssey Infra-red Imager.

Co-Immunoprecipitation analysis. Cells (100 mm dish) were harvested in ice-cold PBS and lysed in hypotonic lysis buffer (20 mM Tris pH7.4, 10 mM KCl, 10 mM $MgCl_2$, 2 mM EDTA, 10% glycerol, 1% Triton, protease inhibitor cocktail (Roche), 0.2 mM PMSF, PhosphStop (Roche)) for 10 minutes before adding NaCl to final concentration 420 mM and rotating overnight at 4°C. Lysates were sonicated briefly before removing insoluble material by centrifugation. An equal volume of hypotonic lysis buffer was added to the lysate to lower the NaCl concentration to 210 mM. 4% of the lysate was

removed for input. 4 μg antibody anti-PARP1 (Santa Cruz #sc-25780), anti-NFI (Santa Cruz #sc-5567), anti-p400 (Bethyl labs #A300-541A), anti-INO80 (Abcam #118787) or non-specific IgG (Millipore #12-370) and 20 μl magnetic Protein G beads were incubated with the lysates for 4 hours at 4°C. Beads were then washed three times with hypotonic lysis buffer containing 200 mM NaCl and twice with 1xTE.

Supplementary References

Patel AG, Flatten KS, Schneider PA, Dai NT, McDonald JS, Poirier GG, Kaufmann SH (2012) Enhanced killing of cancer cells by poly(ADP-ribose) polymerase inhibitors and topoisomerase I inhibitors reflects poisoning of both enzymes. *J Biol Chem.* **287:** 4198-4210.

Supplementary Figure legends

Fig. S1. Inducible PARP1 binding and *FOS* activation. (A-D) ChIP analysis of PARP1 (A and D), NFI (B) or ELK1 (C) bound to the *FOS* promoter in HeLa cells starved in serum-free DMEM (0) or stimulated with PMA for the indicated times (A-C) or in MCF10A cells starved of EGF (-) or stimulated with EGF for 10 minutes (+) (D). Data are presented as means ±SEM ($n \geq 4$) and are the average of at least two independent experiments performed in at least duplicate. (E and F) Graphs show average knockdown achieved for each of the indicated siRNAs in HeLa cells relative to expression in the presence of control siRNAs against *GAPDH* (taken as 100%). Data are presented as means ± SD ($n \geq 3$). (G) Western blot showing the effect of siRNA-mediated depletion of PARP1 and PARP2 on expression of the respective proteins in HeLa cells. (H) Western blot (WB) analysis of FOS protein levels in HeLa cells after incubation with PMA for the indicated time points in the presence of either control *GAPDH* or *PARP1* siRNAs. ELK1 levels were monitored as a loading control. (I) Western blot (WB) of Fos protein induction in wild-type and Parp1 knockout MEFs alongside expression of MAP kinase pathway activation indicators phospho-Elk1 (P-Elk1) and phospho-ERK (P-ERK). Total ERK and Elk1 levels are also shown. Cells were starved in serum-free DMEM (0) or stimulated with PMA for the indicated times. (J) Western blots (WB) of PAR levels (top panels) and PARP1 levels (bottom panels) in HeLa cells following treatment with the indicated concentrations of the PARP1 inhibitors 3AB (left) and KU0058948 (right) in cells stimulated with PMA for 15 mins. Concentrations used throughout this study are indicated by the boxes. The major PARylated species migrating above 130 kDa most

likely represent PARylated PARP1 as reported by others. (K) Real-time RT-PCR measurement of *FOS* mRNA levels in HeLa cells after incubation with PMA at the time points shown and, where indicated, pretreated for 30 minutes with PARP1 inhibitors 3AB, or KU0058948. Data in (F and H) are presented as means ± SD (n = 3) and are representative of at least two independent experiments performed in triplicate. (L) Western blot (WB) showing effect of PARP1 inhibition on FOS protein induction in HeLa cells treated with PMA for the indicated times. Levels of ELK1 and phospho-ELK1(P-ELK1) and phospho-ERK (P-ERK) are also shown. PARP1 inhibition has minimal effects on MAP kinase pathway activity indicators phospho-ELK and phospho-ERK. (M) Western blot analysis of Parp1 expression in wild-type (WT) MEFS or *Parp1-/-* MEFs reconstituted with WT or E988K mutant version of PARP1 where indicated.

Fig. S2. The role of PARP1 in NFI binding to the *FOS* promoter. (A) Western blots showing the effect of removal of Parp1 in knockout MEFs on p300 and NF1 levels throughout the indicated timecourse of PMA induction. (B) ChIP showing the association of NFI with the *FOS* promoter upon PMA stimulation for 10 mins in HeLa cells. Where indicated, cells were treated with the PARP inhibitor PJ34 for 30 minutes before PMA stimulation.

Fig. S3. PARP1 is required for ERK-mediated changes in the chromatin structure in the *FOS* promoter. (A) ChIP showing promoter-specific association of H3K9ac. HeLa cells were treated with either siGAPDH (-) or siPARP1 (+) and serum-starved before treating with PMA. A negative control region was tested 3000 bp upstream of

transcription start site. Binding is calculated relative to H3 ChIP. PCR was performed with primers centred -130 or -3000 bp upstream from the *FOS* TSS. Data are presented as means ± SEM ($n \geq 4$) and are the average of two independent experiments performed in duplicate. (B) Chromatin accessibility measured by real-time PCR (CHART-PCR) at the *FOS* promoter -1 nucleosome. HeLa cells were serum starved or treated with PMA for 10 min. Isolated nuclei were incubated with limiting amounts of DNaseI and the relative levels of nuclease access to the nucleosome were measured by real-time PCR. Percentage access was calculated relative to undigested chromatin DNA. (C) CHART-PCR analysis of the *FOS* promoter -1 nucleosome (black bars) and at a locus centred -3000 bp from the TSS (grey bars). HeLa cells were serum starved or treated with PMA for 10 min. Isolated nuclei were incubated with the indicated units of DNaseI and the relative levels of nuclease access to the nucleosome were measured by real-time PCR. Percentage protection was calculated relative to undigested chromatin DNA (taken as 100% at each locus). Data are presented as means ± SEM ($n = 2$) and are representative independent experiments. (D) Chromatin accessibility measured by real-time PCR (CHART-PCR) at the *FOS* promoter -1 nucleosome. HeLa cells were treated with PMA for 10 min. Cells were treated with siPARP1 or siGAPDH (ctrl) before stimulation. Isolated nuclei were incubated with limiting amounts of DNaseI and the relative levels of nuclease access to the nucleosome were measured by real-time PCR. Percentage access was calculated relative to undigested chromatin DNA. Data are presented as means ± SD ($n = 2$) and are representative of three independent experiments.

Fig. S4. Analysis of H2A.Z binding at the -1 nucleosome. (A) ChIP showing levels of H2A.Z bound to the -130 and -3000 regions of the *FOS* gene relative to the TSS in quiescent HeLa cells. ChIP with non-specific IgG gives an estimation of background signals at the loci. Binding is calculated relative to H3 ChIP. (B) Mononucleosome ChIP assays using H2A.Z, H3 and control IgG antibodies to detect binding to the *FOS* -1 nucleosome. Data are presented as means ± SD (n = 2) and are representative of four independent experiments. (C) Example of a mononucleosomal chromatin preparation, run on an agarose gel and stained with ethidium bromide. The majority of the DNA migrates around 150 bp, indicative of mononucleosomal DNA. (D-F) Graphs show the average knockdown achieved for each of the indicated siRNAs relative to expression in the presence of control siRNAs against *GAPDH* (taken as 100%). Data are presented as means ± SD (n ≥ 3). (G-I) Western blot analysis of H2A.Z (G), p400 (H) and INO80 (I) expression in cells treated with control (ctrl) non-targeting siRNAs or siRNAs targeting the respective gene products. (J) ChIP showing association of NFI with the indicated regions of the *FOS* gene. HeLa cells were serum-starved and where indicated, cells were pre-treated with siRNA directed toward either GAPDH or H2A.Z. (K) Tiling ChIP analysis of changes in H2A.Z enrichment across the *FOS* gene after MAP kinase pathway activation reveals reciprocal PMA-inducible changes in H2A and H2A.Z binding. HeLa cells were serum-starved (top) or starved and treated with PMA for 10 minutes (bottom). H2A (left axis, black lines) and H2A.Z (right axis, grey lines) binding were determined by ChIP and the data presented relative to H3 levels at each position. A diagrammatic illustration of the *FOS* locus is shown below the graphs to illustrate the locations of each

amplicon (grey boxes represent exons). The promoter region showing dynamic changes in H2A and H2A.Z binding is boxed.

Fig. S5. The role of PARP1 in H2A.Z binding to the FOS promoter. (A) ChIP analysis of the indicated histones in serum starved and PMA treated (10 mins) MEFs. Data are presented relative to binding levels in serum starved cells (taken as 1). (B and C) ChIP analysis H2A.Z levels at the indicated positions across the *FOS* locus during PMA-induced gene activation. (B) Wild-type (WT) MEFs and *Parp1* knockout MEFs were serum-starved and treated with PMA for 10 minutes. H2A.Z enrichment is shown relative to H2A ChIP. (C) HeLa cells were serum-starved and treated with PMA for the times shown. Where indicated, cells were pre-treated with the PARP inhibitor 3AB for 30 minutes before PMA stimulation. A schematic of the *FOS* locus and the amplified regions is shown. (D) ChIP in wild-type (WT; black lines) or *Parp1* knockout (grey lines) MEFs showing the relative binding of H2A.Z and H2A to the *p21* promoter at the indicated times after doxyrubicin treatment. Data are presented as a ratio of binding signal (ratio in WT MEFs in the absence of stimulation taken as 1). All data are presented as means ± SEM (n = 2) of two independent experiments. (E) Model of the proposed sequential activation of the *FOS* promoter. Activation of the ERK MAP kinase pathway triggers ELK1 phosphorylation leading to allosteric activation of pre-bound p300, increased local histone acetylation, a change in promoter-proximal nucleosomal structure, and NFI recruitment. PARP1 is then recruited by NFI. H2A.Z is associated with the proximal nucleosome prior to stimulation through the action of p400. Following signal-mediated recruitment, PARP1 promotes the exchange of histone H2A.Z for H2A, at least

in part through affecting p400 binding kinetics. INO80 is important for H2A deposition. This histone exchange establishes a permissive promoter architecture which presumably enables downstream mediator recruitment, the basal machinery (eg TFIIB) and RNA polymerase (PolII) recruitment and engagement, and hence transcriptional activation.

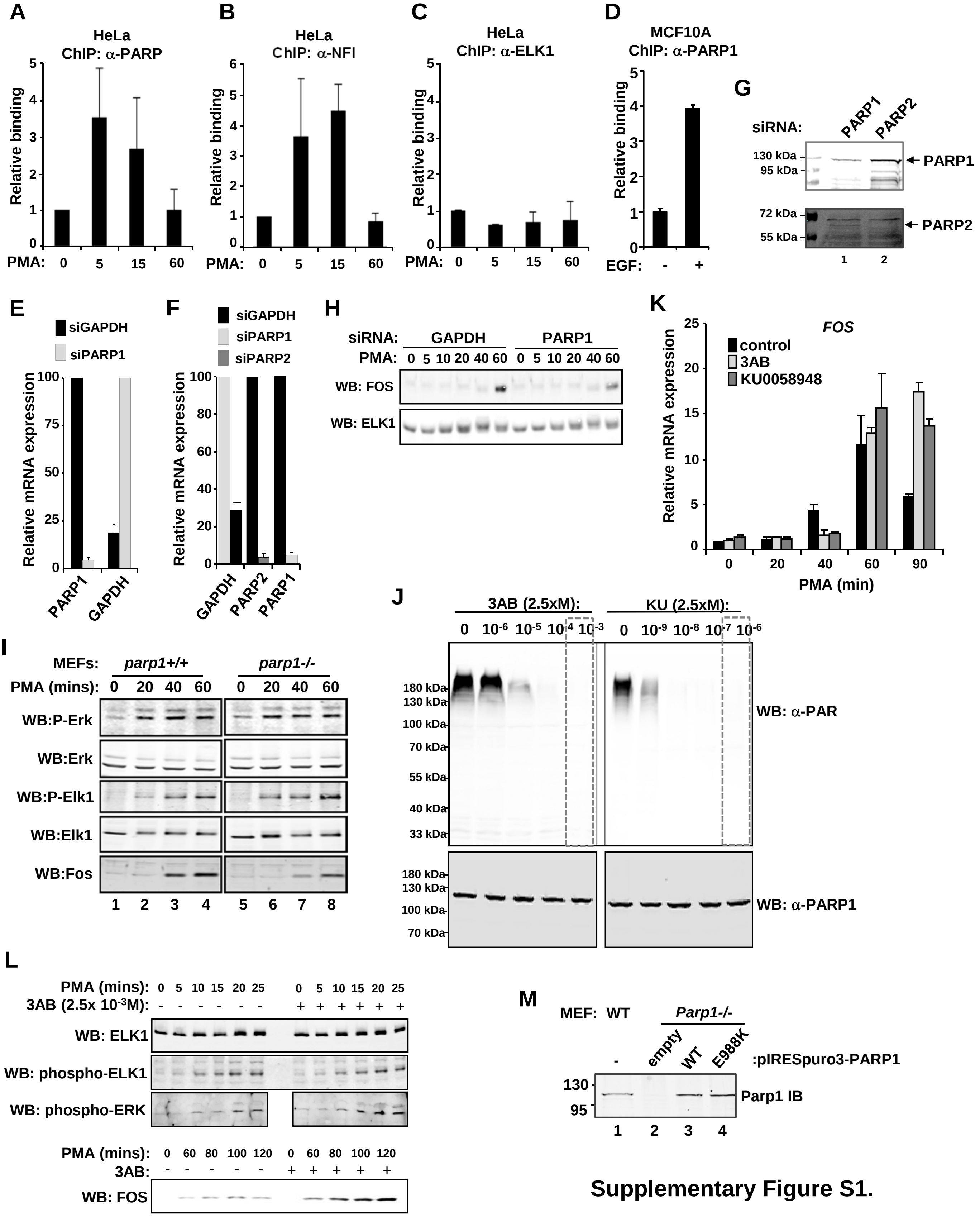

Supplementary Figure S1.

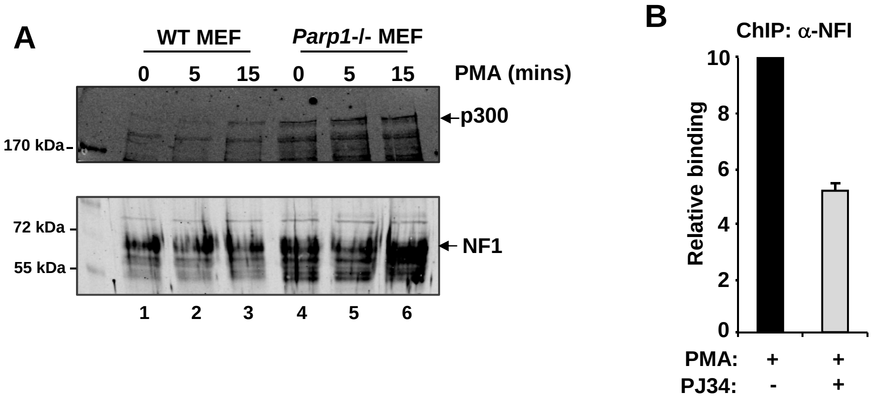

Supplementary Figure S2.

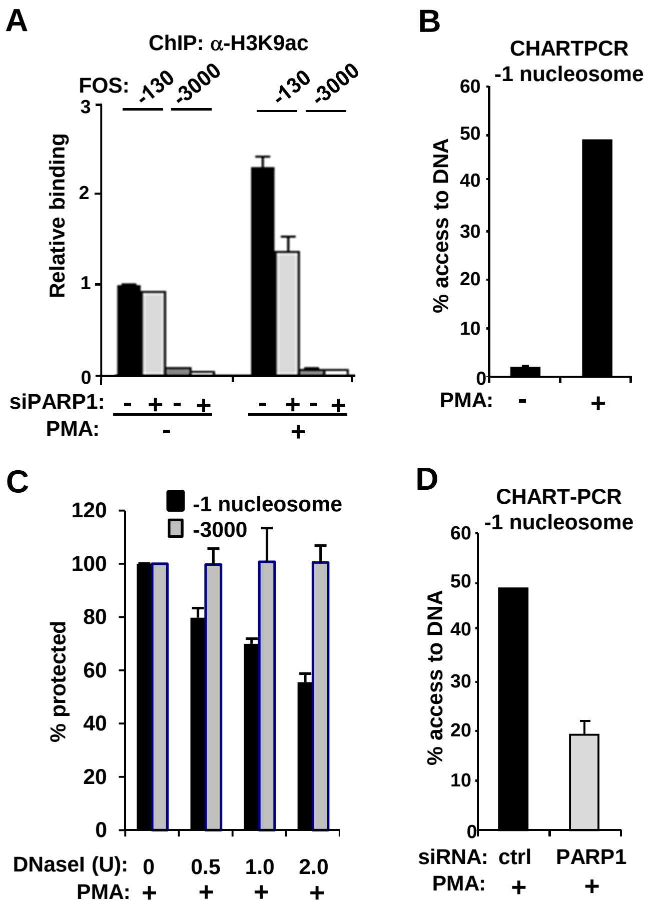

Supplementary Figure S3.

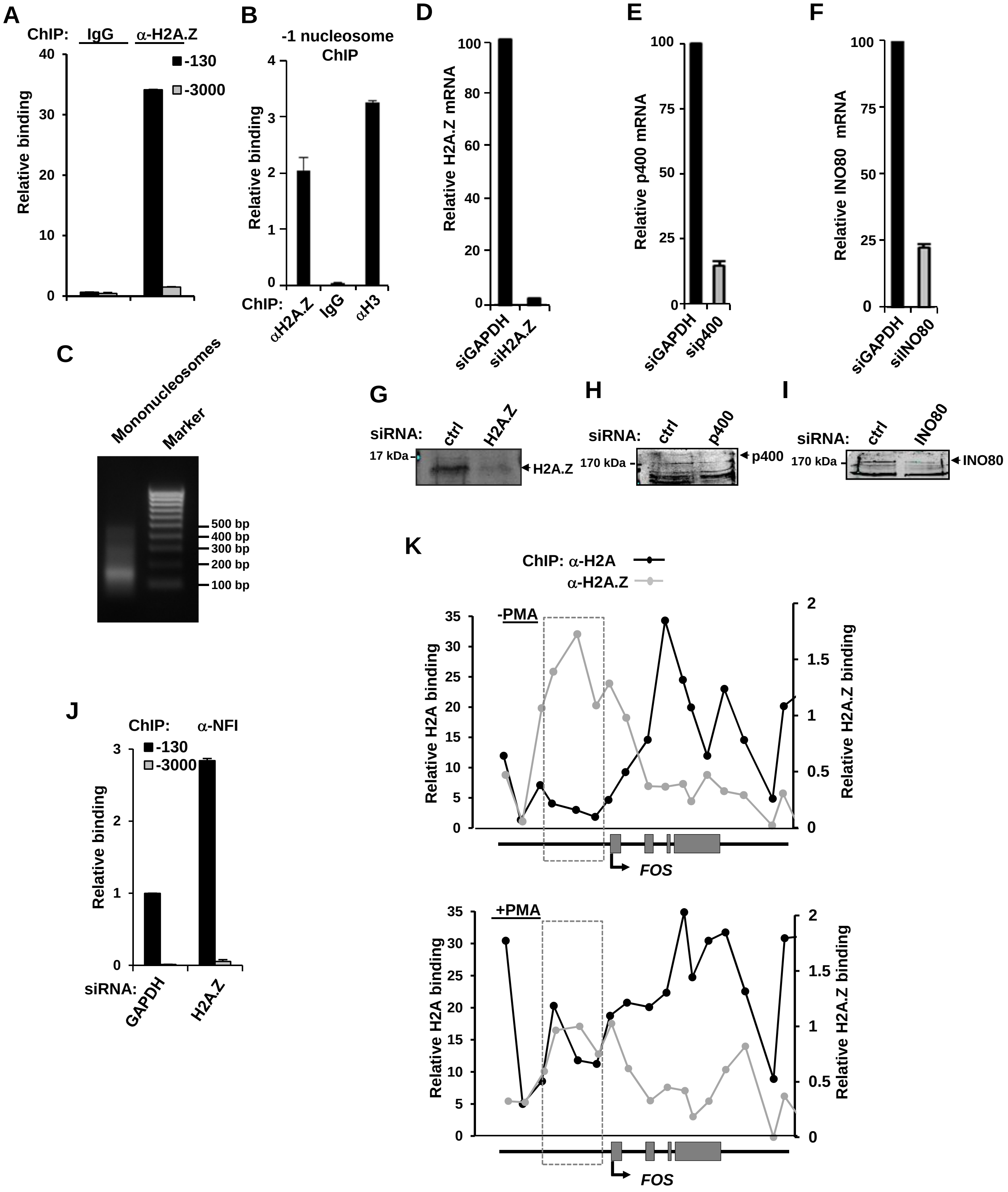

Supplementary Figure S4.

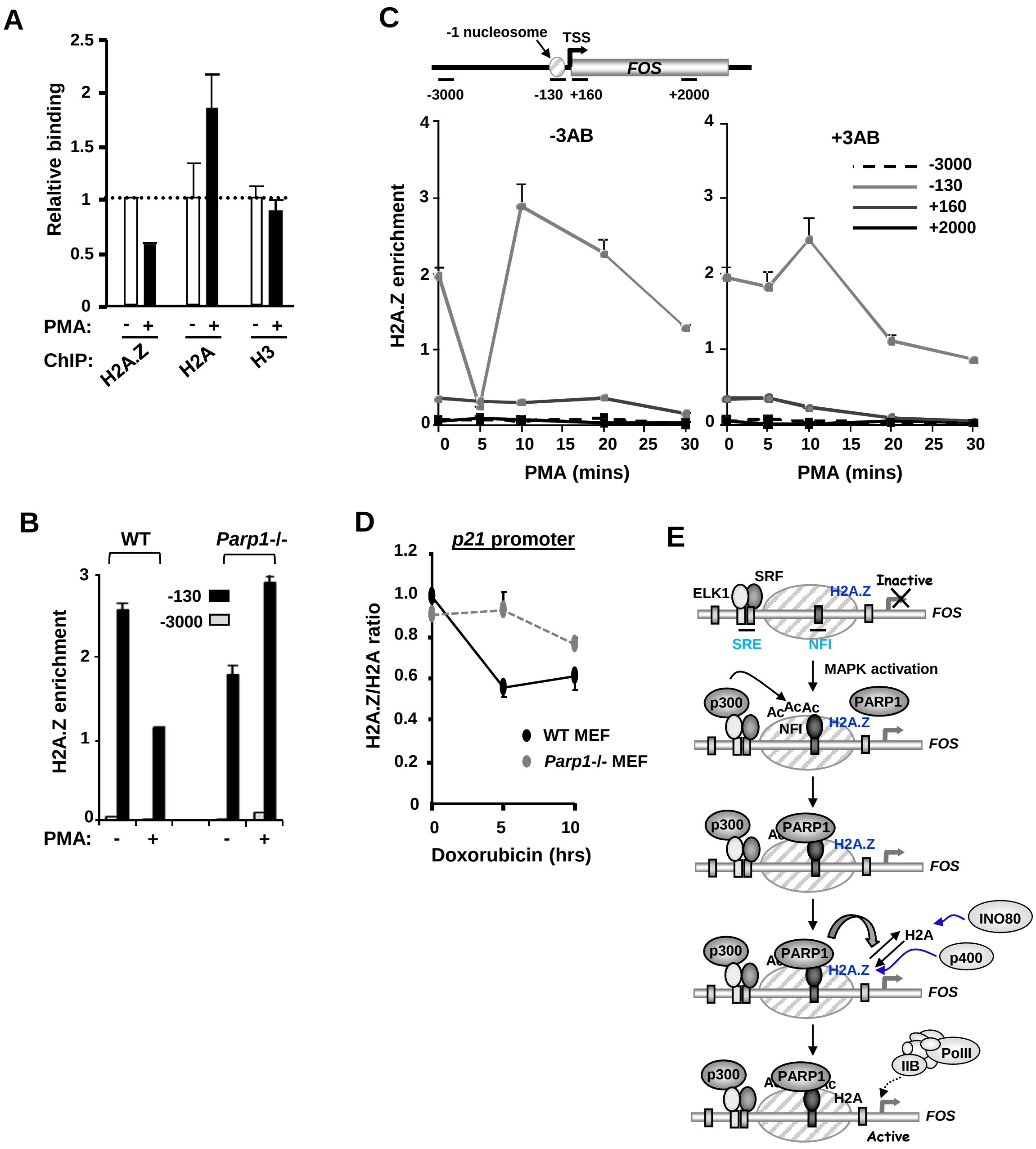

Supplementary Figure S5.
